# Modulation of ESKAPE Bacteria Properties by NK-92 and NK-92-Derived LEVs: First Insights

**DOI:** 10.3390/ijms27093953

**Published:** 2026-04-29

**Authors:** Polina Grebenkina, Elizaveta Tyshchuk, Ananstasia Gulina, Maria Nyukalova, Vladimir Zarubaev, Natalia Arsentieva, Areg Totolian, Lyudmila Kraeva, Dmitry Sokolov

**Affiliations:** 1 Saint-Petersburg Pasteur Institute, St. Petersburg 197101, Russia; grebenkinap@gmail.com (P.G.);; 2 Research Institute of Obstetrics and Gynecology Named After D.O. Ott, St. Petersburg 199034, Russia; 3 Department of Immunology, Pavlov First State Medical University of St. Petersburg, St. Petersburg 197022, Russia; 4 Department of Microbiology, Military Medical Academy Named After S.M. Kirov, St. Petersburg 194044, Russia

**Keywords:** ESKAPE bacteria, LEVs, NK cells, defensin-α1, TLR

## Abstract

ESKAPE pathogens represent a critical threat to global health. This challenge necessitates the development of novel antibacterial strategies. We investigated the antimicrobial potential of NK-92 cells and their derived large extracellular vesicles using flow cytometry, ELISA, confocal microscopy and microbiology assays. Here, we show that both NK-92 cells and NK-92-derived LEVs can interact with bacteria, as confirmed by confocal microscopy and flow cytometry. This interaction is associated with inhibition of colony formation. A possible mechanism can involve defensin-α1 secreted by NK-92 and packed in their LEVs. NK-92-derived LEVs can modulate *S. aureus* viability, colony growth and clindamycin susceptibility. These findings suggest NK cell-derived LEVs as promising strategies to combat multidrug-resistant bacterial infections.

## 1. Introduction

ESKAPE pathogens (*Enterococcus faecium*, *Staphylococcus aureus*, *Klebsiella pneumoniae*, *Acinetobacter baumannii*, *Pseudomonas aeruginosa*, and *Enterobacter* spp.) represent a critical threat to global health. The rapid evolution of resistance mechanisms in these organisms necessitates the development of novel therapeutic agents [1]. Consequently, intensive efforts are focused on discovering new antibacterial strategies [2,3].

A promising option for combating antibiotic-resistant bacteria may be the use of natural killers (NK cells). Their surface expresses receptors capable of interacting with bacterial cells or their components, such as TLR2 [4,5], TLR4 [4], and TLR5 [5,6] (Toll-like receptor). Their proteome also contains substances with antibacterial activity [5,7,8,9,10]. In clinical practice, it seems likely to use NK-92 cells that reproduce all the features of NK cells. They were isolated in 1992 from blood cells of a patient with lymphoma, and in 1998, they were placed in the American Collection of Cell Cultures (ATCC), after which they were widely used for research purposes. Moreover, their clinical application is advancing, with Phase II trials currently investigating NK-92-based therapies for oncology patients [11,12,13].

NK cells secrete extracellular vesicles, including large extracellular vesicles (LEVs) [14]. A defining characteristic of NK cell-derived LEVs is the presence of molecules that facilitate their interaction with cells [15], which provides the possibility of “targeted” delivery of substances using them. Some effects of EVs derived from NK cells on eukaryotic cells are shown: they regulate endothelial cell migration and activation [16], drive macrophage polarization toward a pro-inflammatory phenotype [17], and induce tumor cell death [14]. However, the impact of NK cell-derived LEVs on prokaryotic cells remains unexplored, and data regarding the expression of bacterial-interacting receptors on LEV surfaces are currently lacking.

It is hypothesized that LEVs may be involved in enhancing the body’s reactions against various pathogens. For instance, epithelial cell-derived EVs have been shown to inhibit *Candida albicans* growth [18]. Similarly, patients with bacteremia exhibit elevated levels of neutrophil-derived LEVs in serum, which demonstrated bacterial growth inhibition in experimental models [19]. While analogous effects have not been described to date for NK cell-derived LEVs, their potential antibacterial activity is plausible given the presence of defensin proteins within their composition [9].

The unique proteomic and phenotypic profile of NK cells and their LEVs suggests a capacity for interaction with prokaryotic cells, positioning them as promising candidates for antibacterial therapy. However, their bactericidal and bacteriostatic properties, as well as their ability to modulate bacterial sensitivity to antibiotics, remain uncharacterized. This study aims to elucidate the antimicrobial effects of NK-92 cells and their derived LEVs, focusing on their characteristics that mediate bacterial interaction.

## 2. Results

### 2.1. Evaluation of TLR2 and TLR5 Expression on NK-92-Derived LEVs

**Figure 1:**
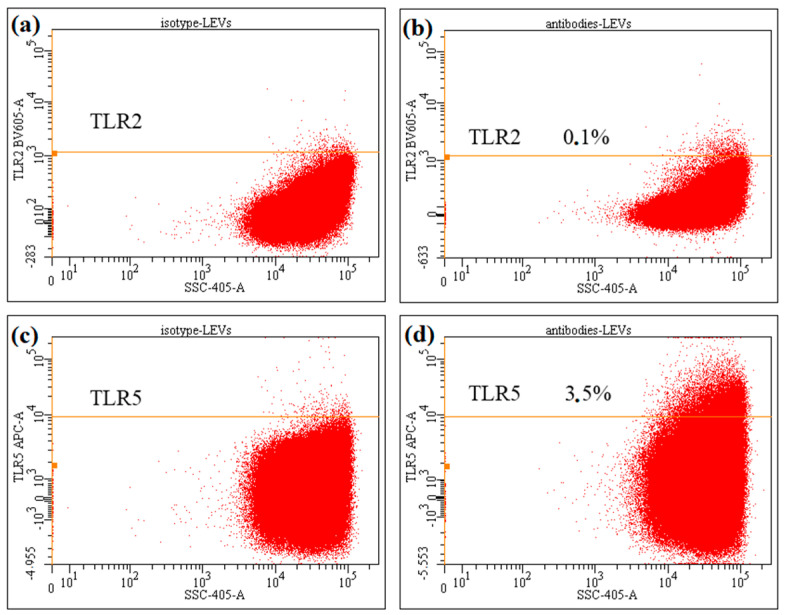
Expression of TLR2 and TLR5 on NK-92-derived LEVs. (**a**,**c**) LEVs stained with isotype control antibodies; (**b**,**d**) LEVs stained with antibodies against TLR2 (**b**) and TLR5 (**d**).

Building on our previous demonstration that NK-92 cells express TLR2 and TLR5 [20], we next assessed the presence of these receptors on NK-92-derived LEVs: analysis revealed that they lack detectable TLR2 expression, while exhibiting only minimal TLR5 signal (Figure 1).

### 2.2. Quantification of α-Defensin-1 Content in the Secretome of NK-92 Cells and Their Derived LEVs

**Figure 2:**
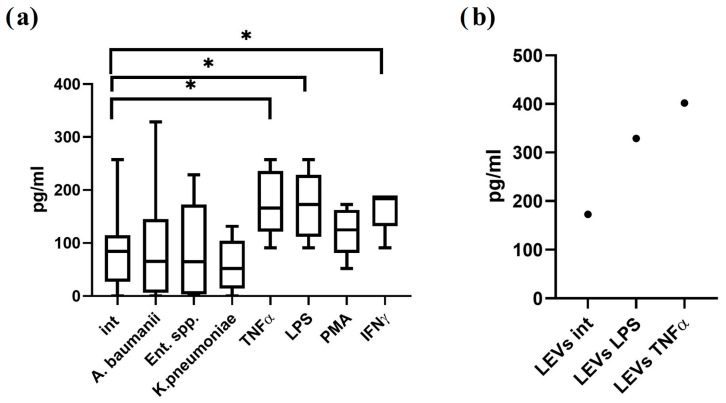
Quantification of α-defensin-1 levels in conditioned media from NK-92 cells (**a**) and in NK-92-derived LEVs (**b**). Studied conditioned media from unstimulated NK-92 cells (Int) or cells stimulated with bacterial supernatants (*A. baumannii*, *Enterobacter* spp., *K. pneumoniae*) or inflammatory agonists (TNF-α, LPS, PMA, IFN-γ) (n = 4 with 2 technical replicates). LEVs are derived from unstimulated (LEVs int), LPS-(LEVs LPS), or TNF-α-stimulated (LEVs TNF-α) NK-92 cells (summary n = 3). * *p* < 0.05.

α-defensins and β-defensins are cationic antimicrobial peptides with pore-forming activity that have been previously identified in the proteome of NK cells and their extracellular vesicles by flow cytometry and mass spectrometry [5,9]. In this study, we employed enzyme-linked immunosorbent assay (ELISA) to quantify α-defensin-1 levels in conditioned media from NK-92 cells and in NK-92-derived LEVs. We demonstrate that NK-92 cells constitutively secrete α-defensin-1 (Figure 2a). Stimulation with LPS, TNF-α, or IFN-γ significantly enhanced α-defensin-1 production relative to unstimulated controls. Furthermore, we assessed α-defensin-1 content in LEVs isolated from NK-92 cells cultured under varying conditions. The peptide was detected in LEVs derived from both unstimulated NK-92 cells and those stimulated with LPS or TNF-α (Figure 2b).

### 2.3. NK-92 Cells Affect Colony Formation and Growth of ESKAPE Pathogens

**Figure 3:**
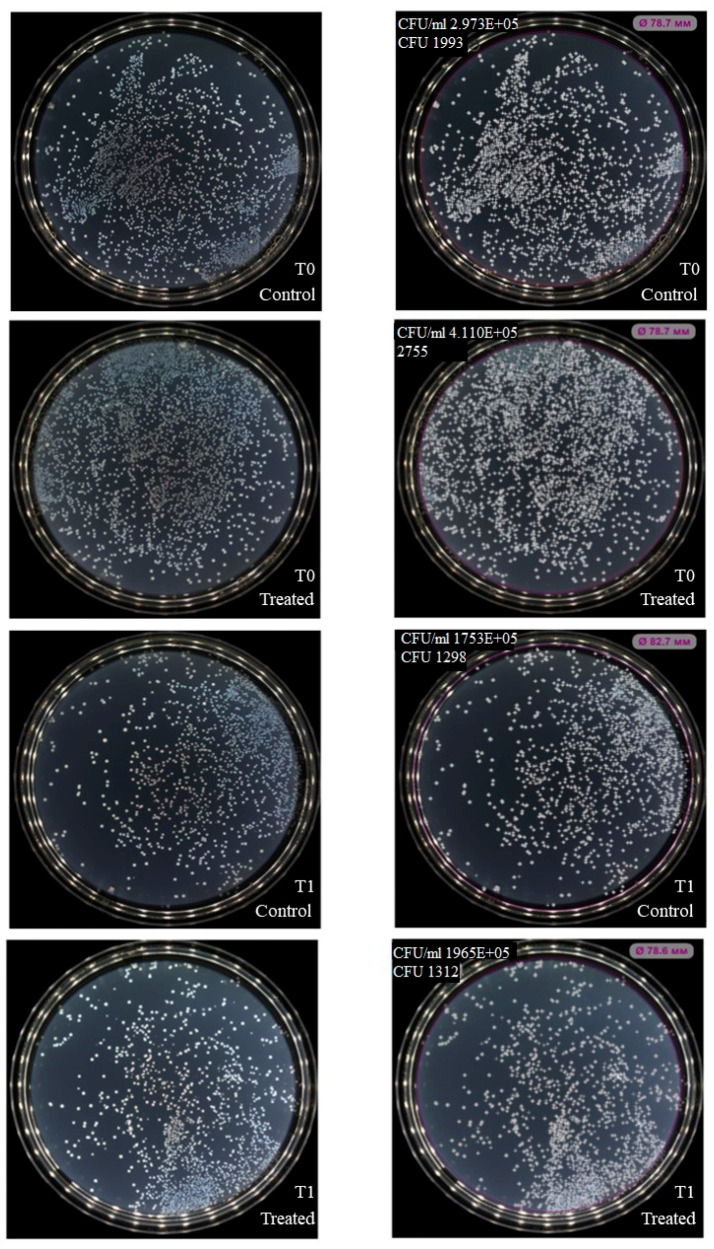
Effect of NK-92 cells on *E. faecium* colony formation and growth. Colony formation assay: *E. faecium* plated alone (Control) or co-cultured with NK-92 cells (Treated). (**Left**) Raw plate images; (**Right**) automated analysis (Scan500, Interscience, Saint Nom la Bretèche, France). T_0_/T_1_: plating time points, E + 0n = 10^n^.

**Figure 4:**
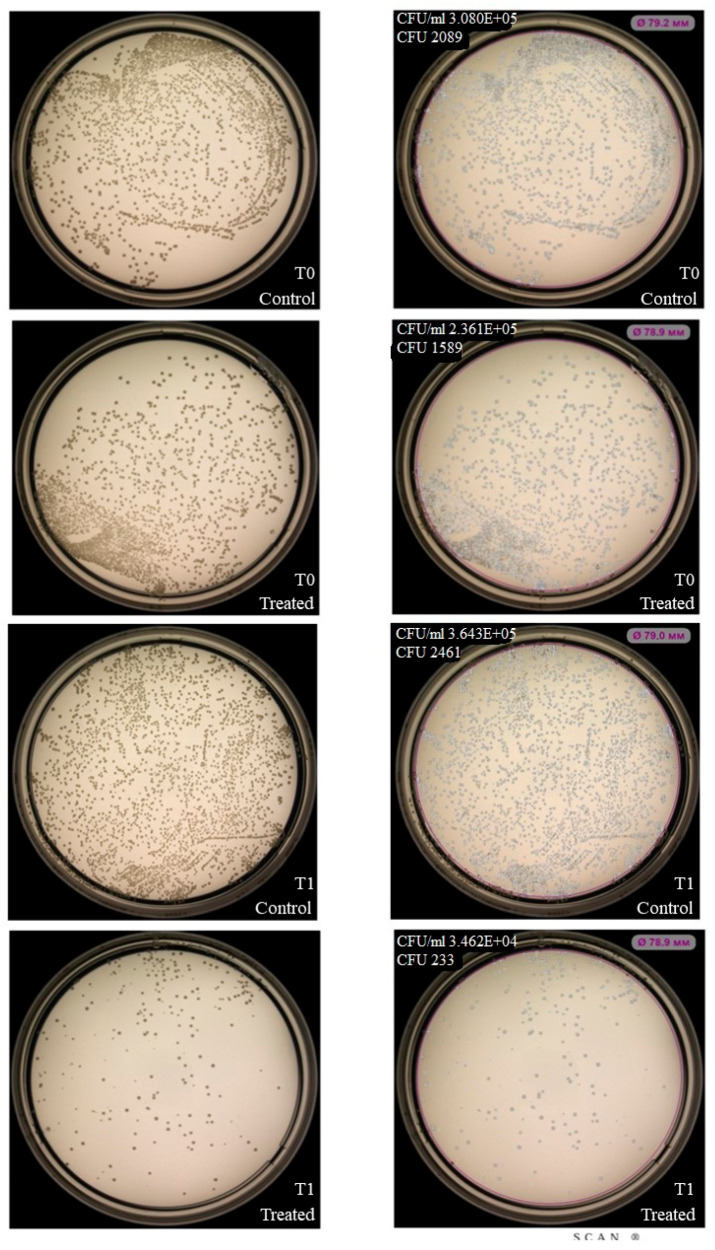
Effect of NK-92 cells on *S. aureus* colony formation and growth. Colony formation assay: *S. aureus* plated alone (Control) or co-cultured with NK-92 cells (Treated). (**Left**) Raw plate images; (**Right**) automated analysis (Scan500, Interscience). T_0_/T_1_: plating time points, E + 0n = 10^n^.

**Figure 5:**
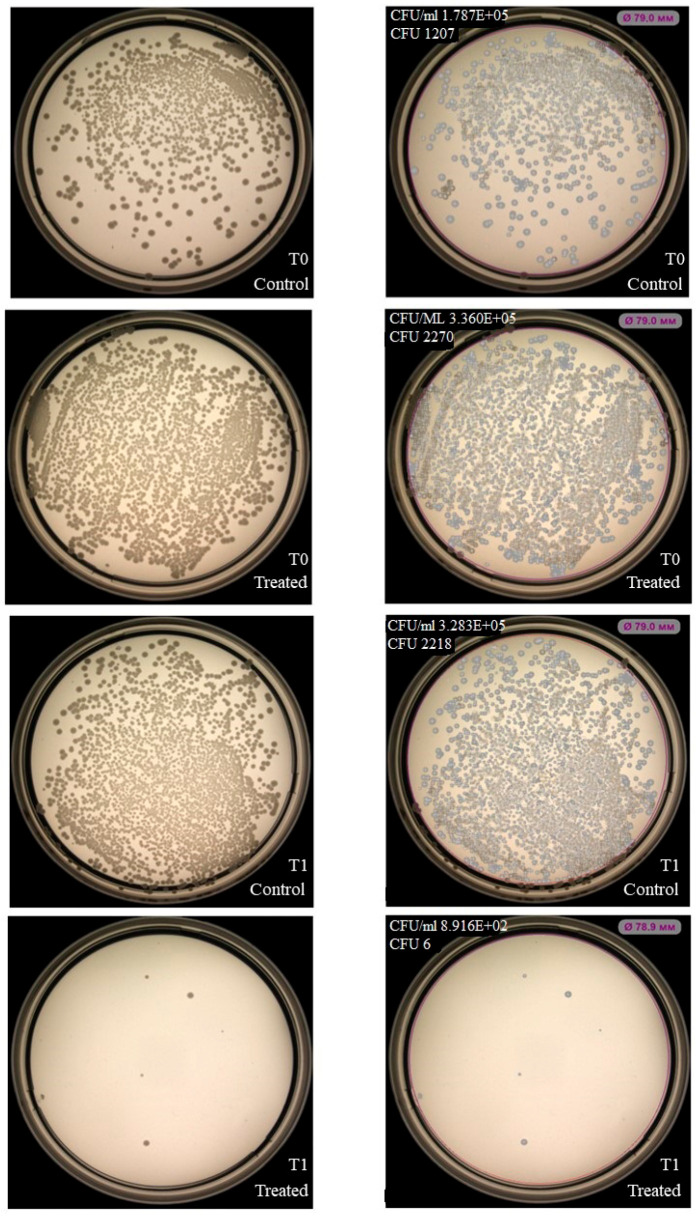
Effect of NK-92 cells on *K. pneumoniae* colony formation and growth. Colony formation assay: *K. pneumoniae* plated alone (Control) or co-cultured with NK-92 cells (Treated). (**Left**) Raw plate images; (**Right**) automated analysis (Scan500, Interscience). T_0_/T_1_: plating time points, E + 0n = 10^n^.

**Figure 6:**
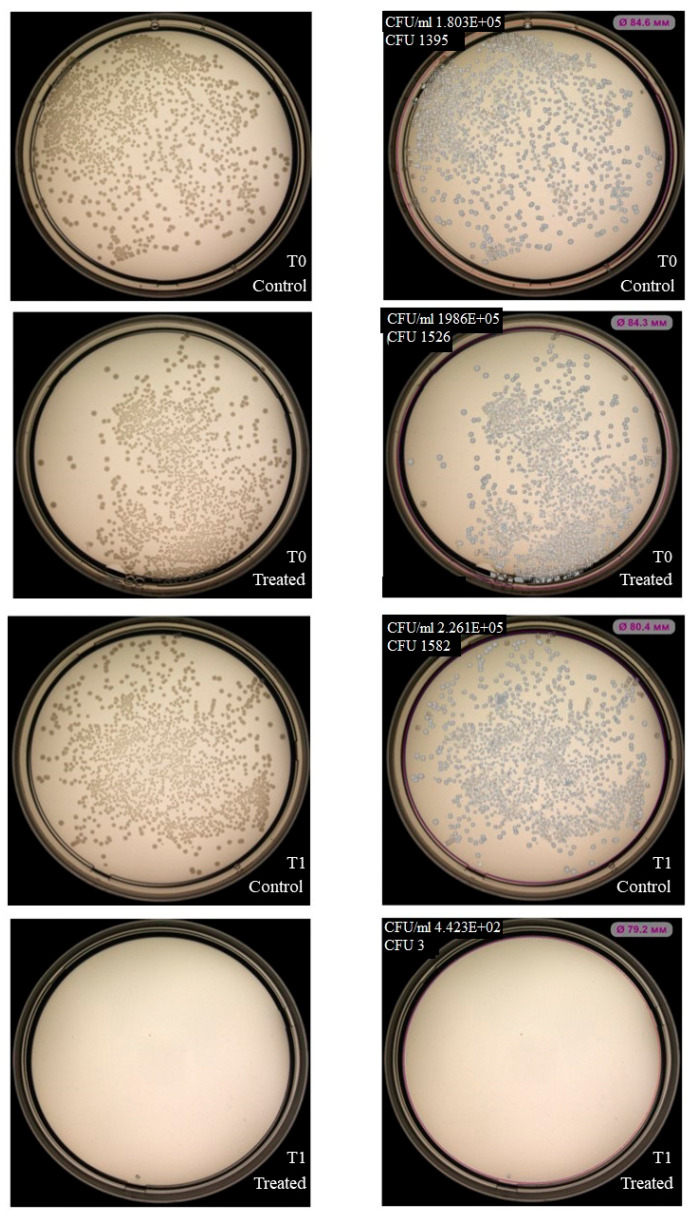
Effect of NK-92 cells on *A. baumanii* colony formation and growth. Colony formation assay: *A. baumanii* plated alone (Control) or co-cultured with NK-92 cells (Treated). (**Left**) Raw plate images; (**Right**) automated analysis (Scan500, Interscience). T_0_/T_1_: plating time points, E + 0n = 10^n^.

**Figure 7:**
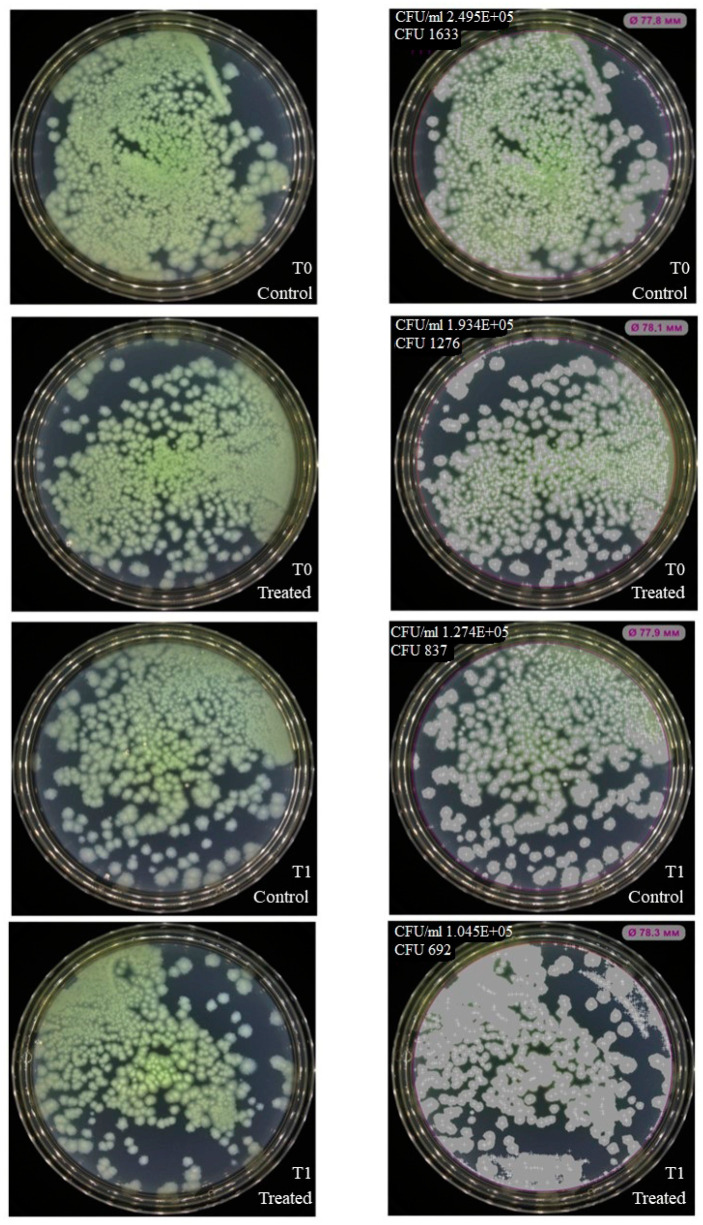
Effect of NK-92 cells on *P. aeruginosa* colony formation and growth. Colony formation assay: *P. aeruginosa* plated alone (Control) or co-cultured with NK-92 cells (Treated). (**Left**) Raw plate images; (**Right**) automated analysis (Scan500, Interscience). T_0_/T_1_: plating time points, E + 0n = 10^n^.

**Figure 8:**
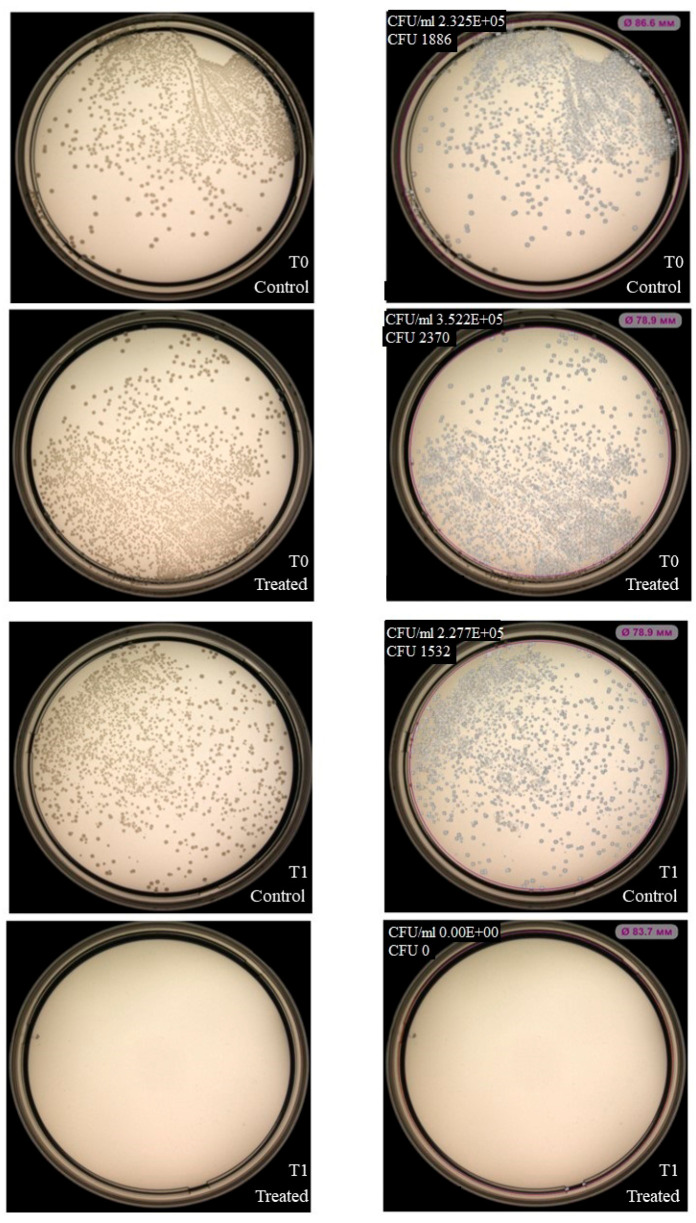
Effect of NK-92 cells on *Enterobacter* spp. colony formation and growth. Colony formation assay: *Enterobacter* spp. plated alone (Control) or co-cultured with NK-92 cells (Treated). (**Left**) Raw plate images; (**Right**) automated analysis (Scan500, Interscience). T_0_/T_1_: plating time points.

NK-92 cells had no inhibitory effect on colony formation of *Enterococcus faecium (*Figure 3), but inhibition was observed for *Staphylococcus aureus*, *Klebsiella pneumoniae*, *Acinetobacter baumannii* (Figure 4, Figure 5 and Figure 6). Under the same experimental conditions colony formation of *Pseudomonas aeruginosa* did not change (Figure 7), while colony formation of *Enterobacter* spp. is also inhibited (Figure 8 and Figures S1–S3).

### 2.4. Confocal Microscopy Visualization of NK-92 Cell Interaction with P. aeruginosa

*P. aeruginosa* was selected as a representative ESKAPE pathogen to characterize the interaction between NK-92 cells and ESKAPE bacteria. Using confocal microscopy, we acquired z-stack optical sections of co-cultured specimens, enabling reconstruction of NK-92/*P. aeruginosa* interfaces (Video S1, Figure S4). Confocal microscopy confirmed direct physical contact between NK-92 cells and *P. aeruginosa*, which proves the possibility of their interaction.

### 2.5. NK-92-Derived LEVs Modulate Colony Formation and Growth of S. aureus and K. pneumoniae

**Figure 9:**
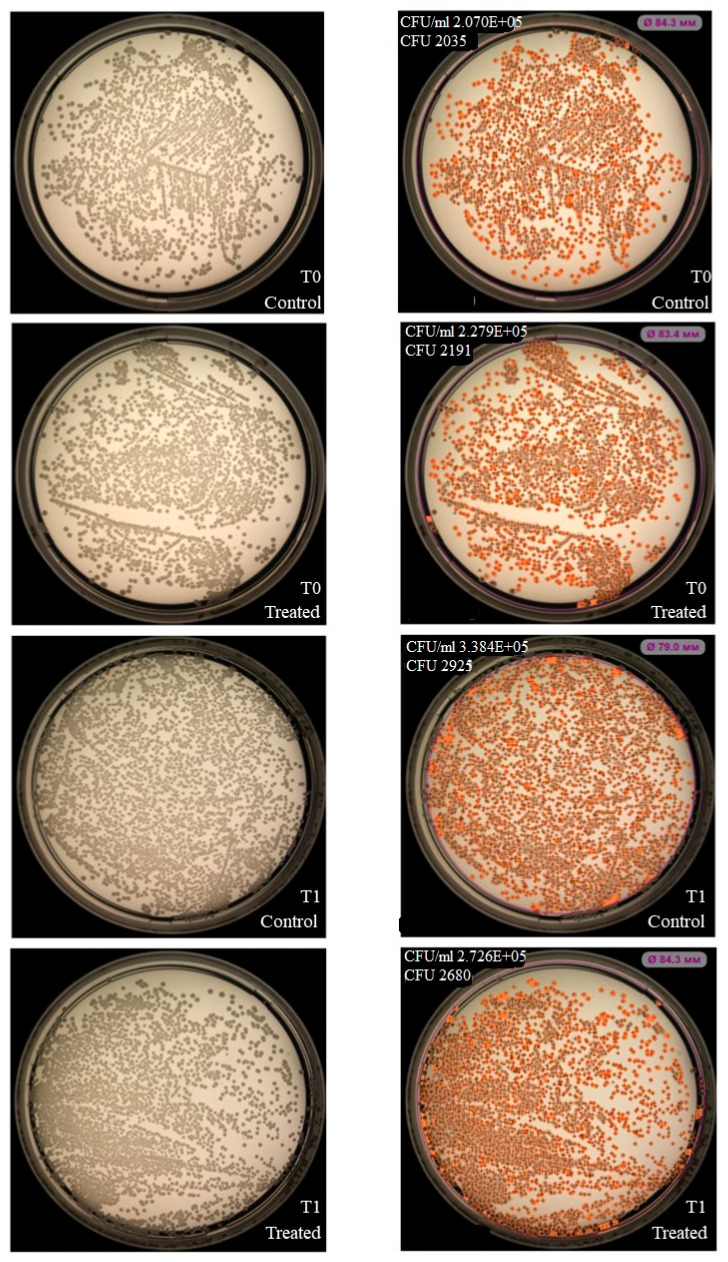
Effect of NK-92-derived LEVs on *K. pneumoniae* colony formation and growth. Colony formation assay: *K. pneumoniae* plated alone (Control) or co-cultured with LEVs (Treated). (**Left**) Raw plate images; (**Right**) automated analysis (Scan500, Interscience). T_0_/T_1_: plating time points, E + 0n = 10^n^.

**Figure 10:**
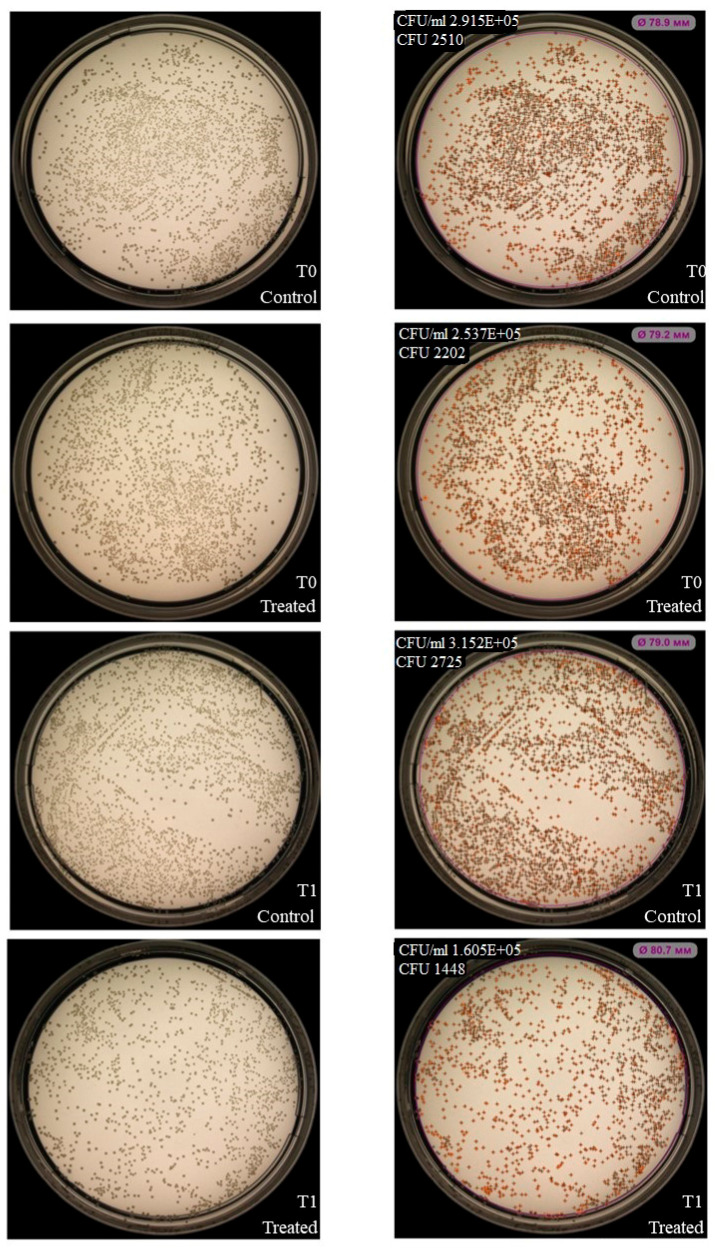
Effect of NK-92-derived LEVs on *S. aureus* colony formation and growth. Colony formation assay: *S. aureus* plated alone (Control) or co-cultured with LEVs (Treated). (**Left**): Raw plate images; (**Right**): Automated analysis (Scan500, Interscience). T_0_/T_1_: plating time points, E + 0n = 10^n^.

Based on prior experimental results and their distinct clinical relevance, we selected *S. aureus* (Gram-positive) and *K. pneumoniae* (Gram-negative) as representative models to evaluate the antimicrobial activity of NK-92-derived LEVs. While LEVs did not affect colony formation or growth of *K. pneumoniae* under the tested conditions (Figure 9), exposure to NK-92-derived LEVs resulted in a reduction in *S. aureus* colony counts following 1 h incubation (Figure 10).

### 2.6. Flow Cytometry-Based Tracking of CFSE Label Transfer from NK-92-Derived LEVs to K. pneumoniae and S. aureus

**Figure 11:**
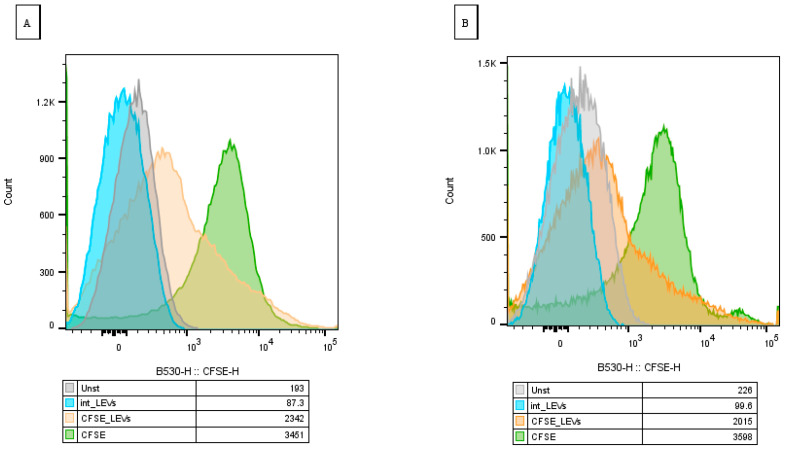
Flow cytometry analysis of CFSE transfer from NK-92-derived LEVs to *K. pneumoniae* (**A**) and *S. aureus* (**B**). Unst—unstained bacteria, CFSE—CFSE-labeled bacteria; int_LEVs—bacteria co-cultured with LEVs derived from unstained NK-92; CFSE_LEVs—bacteria co-cultured with LEVs derived from CFSE-labeled NK-92. The tables show the MFI values.

Flow cytometry analysis demonstrated a significant increase in bacterial fluorescence intensity following co-incubation with CFSE-labeled LEVs for both *K. pneumoniae* (Figure 11A) and *S. aureus* (Figure 11B) compared to controls. These data indicate a physical association of bacterial cells and NK-92-derived LEVs.

### 2.7. NK-92-Derived LEVs Reduce Viability of S. aureus

**Figure 12:**
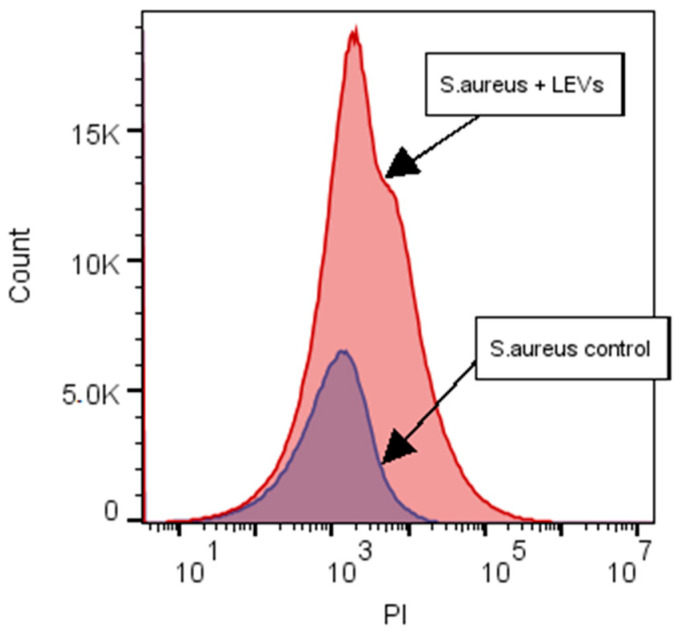
PI-based assessment of *S. aureus* viability following exposure to NK-92-derived LEVs. *S. aureus* control—intact *S. aureus*, *S. aureus* + LEVs—*S. aureus* after exposure to NK-92-derived LEVs.

Although the CFSE label was transferred from NK-92-derived LEVs to both *S. aureus* and *K. pneumoniae*, an inhibitory effect on colony formation was observed only for *S. aureus*. Based on this, we decided to further evaluate the effects of LEVs’ interaction only on *S. aureus* using a kit for determining bacterial viability. We observed a shift in the bacterial population towards PI after interaction with NK-92-derived LEVs relative to intact *S. aureus*, which indicates an increase in the number of dead bacterial cells in the population (Figure 12).

### 2.8. Modulation of Antibiotic Susceptibility in S. aureus by NK-92 Cell-Derived LEVs

To evaluate whether NK-92 cell-derived LEVs modulate bacterial susceptibility to antibiotics, we performed disk diffusion assays using *S. aureus*. Co-incubation of bacteria with NK-92-derived LEVs increased the diameter of inhibition zones for several antibiotics: clindamycin (+5.1 ± 3.0%), erythromycin (+1.2 ± 0.8%), and cefoxitin (+4.8 ± 1.6%) (Table 1). In contrast, no changes in susceptibility were observed for ampicillin/sulbactam, norfloxacin, or benzylpenicillin, irrespective of the baseline resistance phenotype of the *S. aureus* strain. Furthermore, pre-stimulation of NK-92 cells with TNF-α prior to LEVs isolation did not enhance the potentiating effect of LEVs on antibiotic activity.

### 2.9. NK-92-Derived LEVs Reduce the Minimum Inhibitory Concentration of Clindamycin Against S. aureus

**Figure 13:**
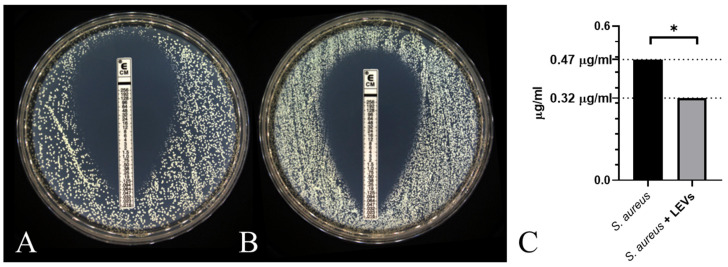
The effect of LEVs on the minimum inhibitory concentration of clindamycin against *S. aureus*. (**A**) Intact *S. aureus*; (**B**) *S. aureus* cultured in the presence of NK-92-derived cells; (**C**) a graph based on 4 experiments with 1 technical repeat, * *p* < 0.05.

Building on the observed potentiation of clindamycin activity in disk diffusion assays, we next assessed whether NK-92 cell-derived LEVs modulate the minimum inhibitory concentration of this antibiotic against *S. aureus*. Using the E test, we observed that co-incubation with NK-92-derived LEVs reduced the MIC of clindamycin by 32% relative to untreated controls: 47 µg/mL to 32 µg/mL (Figure 13).

## 3. Discussion

Opportunistic pathogens within the ESKAPE group of bacteria are leading causes of nosocomial infections, exacerbated by multidrug resistance that severely limits treatment options. This global health challenge necessitates the development of novel antibacterial strategies or adjuvants to potentiate existing antibiotics.

In this study, we investigated the antimicrobial potential of NK-92 cells and their derived LEVs. The therapeutic utility of NK-92 cells is already well-established in oncology, where they demonstrate potent antitumor efficacy without severe adverse effects [11,12]. This, combined with the antimicrobial properties described herein, positions NK-92 cells as promising candidates for repurposing in antibacterial therapy.

NK cells exert cytotoxicity primarily through granulysin and granzymes [7,8]. While traditionally studied in the context of tumor lysis, these effector proteins also exhibit direct antibacterial activity. Granulysin displays pore-forming properties, while granzymes induce reactive oxygen species generation within bacterial cells, leading to cell death [7]. NK-92 cells express antimicrobial α- and β-defensins [5,9] with broad-spectrum activity [10].

In addition to NK-92 cells, we examined the possible antimicrobial effects of their extracellular vesicles. By definition, these are non-multiplying particles limited by a lipid layer that have separated from cells. Since we previously determined the size of these particles (>200 nm) [21], we can describe them as LEVs [22].

A promising strategy to counteract antibiotic resistance involves engineering new systems for drug delivery. Liposome-based nanocarriers are under intensive development due to their favorable safety profile, scalable production, and tunable surface properties that enable targeted delivery. For instance, encapsulation of rifabutin into negatively charged liposomes significantly enhanced its activity against methicillin-resistant *S. aureus* biofilms [23]. Since extracellular vesicles are natural liposomes, their use may be suggested as an alternative.

Some studies show the possible role of EVs in antimicrobial immunity. For instance, EVs derived from the Leuk-1 cells have been shown to inhibit *Candida albicans* growth and induce their morphological alterations in vitro [18]. Notably, even plants use EVs for defense. Upon pathogen challenge, algae modify their EV cargo, enriching the proteome with molecules involved in cell wall remodeling and stress signaling [24]. In addition, some researchers use EV modulation techniques. In this way, EVs were obtained from LPS-induced HepG2 cells, which were then coated with antimicrobial peptide-a, which led to the appearance of an antibacterial effect of EVs compared with intact [25].

The analysis of possible antibacterial effects requires the determination of phenotypic and proteomic features of NK-92 cells and NK-92-derived LEVs.

Receptor interactions are necessary for binding to the bacterial cell, with Toll-like receptors serving as key candidates. While peripheral blood NK cells express TLR2, TLR4, and TLR5 [4,5], we previously confirmed TLR2 and TLR5 expression on NK-92 cells [20]. This receptor profile supports their potential to engage prokaryotic targets. Confocal microscopy confirmed direct physical contact between NK-92 cells and *P. aeruginosa*, which proves the possibility of their interaction.

Surface analysis of NK-92-derived LEVs revealed undetectable TLR2 levels and minimal TLR5 expression. This likely reflects low receptor density on the LEV’s surface, potentially below the flow cytometry detection limit without prior stimulation of parental cells. Nevertheless, since they are expressed on source cells, we can expect the presence of TLRs on NK-92-derived LEVs’ surface, and hence binding to a bacterial cell. The transfer of the fluorescent label from LEVs to bacteria, which we demonstrated, also indicates the interaction of objects.

Using various methods, defensins, cationic peptides with a pore-forming effect, were found among the proteome of NK cells and their LEVs: α-defensins were found in NK cells, while the β-form was found in the composition of the LEVs proteome [5,9]. In this work, we analyzed the content of α-defensin in conditioned media obtained from NK-92 cells and their LEVs. We established that α-defensin is constitutively secreted by NK-92 cells, with levels modulated by various inducers. LPS, a component of the cell wall of Gram-negative bacteria, caused increased defensin secretion, which is consistent with previously obtained results [5]. TLR4 is required for LPS recognition, but we found that it was not expressed on NK-92 cells. It was previously shown that LPS causes activation of NK cells by enhancing IFN-y production [26], and it was also found that LPS can cause activation of NK cells in the presence of IL-2 [27], which confirms that NK cell functionality can be regulated by bacterial components. In our experiments, bacterial supernatants did not change defensin production, which may be caused by the presence of several substances in them that have a multidirectional effect on the defensin production by NK cells. Indeed, pathogens can suppress defensin production via virulence proteins to ensure survival [28], highlighting the complex dynamics of host–pathogen interactions.

We detected α-defensin in LEVs from both unstimulated and stimulated NK-92 cells. The proteome of LEVs can be determined by the conditions of parental cells culturing. Thus, it was shown that PMA and IL-1β stimulation increase perforin content in NK-92-derived LEVs [29]. Consequently, optimizing NK-92 culture conditions with specific inducers represents a promising strategy to enrich LEVs with defensins and enhance their bacteriostatic potential.

Defensins exhibit broad-spectrum activity against ESKAPE pathogens, including antibiotic-resistant strains of *K. pneumoniae* [30], *S. aureus* and *P. aeruginosa* [31]. In 2022, a study demonstrated that an α-defensin-based agent exhibited bactericidal properties against multidrug-resistant *K. pneumoniae*, *P. aeruginosa*, *A. baumannii*, and *S. aureus*; the mechanism may involve interaction with bacterial surface components [32].

The presence of TLR2 and TLR5 on the membrane, which provide binding to the bacterial cell, as well as the presence of antibacterial proteins in the proteome, positions NK cells and their derived vesicles as promising candidates for novel antibacterial therapies. These mechanisms likely provide antimicrobial effects observed in this study.

In 1989, the antibacterial potential of NK cells was recognized: it was demonstrated that they interact with bacteria via membrane contact rather than phagocytosis, and that NK cell supernatants actively inhibit bacterial colony growth [33]. Despite this early discovery, the direct bactericidal function of NK cells remains relatively underexplored compared to their well-characterized immunoregulatory roles in coordinating bacterial elimination by macrophages and other immune effectors. Recently, a 2023 study detailed the antibacterial activity of NK cells against *Staphylococcus aureus* and *Pseudomonas aeruginosa*, reporting increased bacterial death upon co-culture. Notably, an effector:target ratio of 200:1 proved most effective for peripheral blood NK cells after 4–6 h, whereas an E:T ratio of 25:1 was sufficient for NK-92 cells over the same duration [34]. In our experiments, a lower E:T ratio (10:1) with only 1 h co-incubation effectively suppressed colony formation of *S. aureus*, *K. pneumoniae*, *A. baumannii*, *Enterobacter* spp., though no effect was observed for *E. faecium* or *P. aeruginosa*. This indicates the need for further study of the interaction of NK cells with these bacteria.

For further LEVs experiments, we focused on *S. aureus* and *K. pneumoniae* as representative models of Gram-positive and Gram-negative pathogens, respectively. While the bactericidal or bacteriostatic properties of NK cell-derived LEVs have not been previously characterized, analogous effects have been reported for neutrophil-derived EVs [19] and macrophage-derived EVs secreted during infection, which contain iron-regulating proteins that inhibit bacterial growth [35]. In our work, it was found that the fluorescent label from LEVs is transmitted by both *S. aureus* and *K. pneumoniae*, but the inhibitory effect of LEVs on colony growth is shown only for *S. aureus*. This differential susceptibility may be attributed to the structural features of the bacterial surface. *K. pneumoniae* is a Gram-negative pathogen with a protective capsule [36], which can provide low permeability for substances from LEVs into the cell, despite membrane interaction. But this suggestion needs more experimental evidence.

To further evaluate the effect of LEVs on bacteria, their effect on *S. aureus* was analyzed, since a bacteriostatic effect was shown for this ESKAPE bacterium. To assess the effect on bacterial viability, the PI dye, which penetrates into dead cells, was used. We demonstrated an increase in the intensity of fluorescence along the PI detection channel in the *S. aureus* population cultivated in the presence of LEVs, compared with the intact population. This indicates LEVs-induced loss of membrane integrity and reduced bacterial viability. We hypothesize that this effect may be mediated by defensins and cytotoxic proteins such as granulysin [7,10] packaged within LEVs.

Building on these observations, we assessed whether NK-92-derived LEVs can modulate antibiotic susceptibility in *S. aureus*. Initial screening via disk diffusion assay was evaluated with six clinically relevant antibiotics. Among these, clindamycin, a lincosamide antibiotic, is an alternative therapy for methicillin-resistant *S. aureus* infections. Given emerging reports of reduced clindamycin susceptibility [37], strategies to potentiate its activity are clinically valuable. Although proteomic analysis of NK-92-derived LEVs did not identify canonical protein synthesis inhibitors [9], we observed a reduction in the minimum inhibitory concentration of clindamycin in the presence of LEVs. This effect may be explained by pore-forming defensins [38] or granulysin [39] delivered by LEVs: upon membrane interaction, these peptides could compromise bacterial envelope integrity, thereby enhancing clindamycin access to its intracellular target. Further studies are required to elucidate the underlying mechanisms.

We also explored whether pre-stimulation of parental NK-92 cells with TNF-α could enhance LEV antimicrobial activity, based on prior evidence that cytokine exposure increases cytotoxic components in EVs [29]. However, LEVs derived from TNF-α-stimulated NK-92 cells did not exhibit antibiotic-potentiating effects against *S. aureus* under our experimental conditions. This suggests that alternative inducers are required to enhance LEV-mediated antibacterial functions, which can be a new aim for future investigation.

However, several limitations of this study should be acknowledged. We used ATCC strains, which limits the extrapolation of our findings to clinically relevant isolates and does not reflect genetic variability and adaptation in natural bacterial populations. Additionally, certain experiments were conducted with a limited number of replicates, as these stages served primarily for preliminary screening. The disk diffusion assay revealed only minor changes in inhibition zone diameters upon LEVs exposure; these results were therefore used as a screening step to select the antibiotic whose MIC would be evaluated under LEVs treatment. Although elucidating ligand–receptor interactions between NK-92 cells/LEVs and bacteria via TLRs was not the primary aim of this study, the presence of these receptors suggests their potential involvement. Furthermore, modulating LEVs’ properties or increasing their production will likely be necessary to achieve a strengthening of the antibacterial effect. It should be noted that this study is a pilot project. This is the first work to evaluate both NK cells and their LEVs as promising modulators of ESKAPE bacteria characteristics. Our findings clearly demonstrate the presence of TLRs and defensins on the effectors, confirm the transfer of fluorescent labels from LEVs to bacteria, and reveal LEVs-induced changes in the clindamycin MIC for *S. aureus*. The comprehensive dataset generated through multiple methodological approaches establishes a clear direction for future research, including studies with increased replicates, testing on clinical and field isolates with expanded sampling, and employing advanced techniques to elucidate the underlying mechanisms of the observed phenomena.

In summary, the co-expression of bacterial-binding receptors (TLR2, TLR5) and antimicrobial effectors (defensins, granulysin) makes NK-92 cells and their derived LEVs promising candidates for novel antibacterial strategies. However, translational application faces practical challenges, including the relatively low yield of LEVs from NK-92 cultures and the concentration of bioactive components inside. Future work could focus on optimizing parental cell culture conditions and LEV engineering approaches to potentiate vesicle production and enrich antimicrobial payload. These steps will help to develop NK-92-derived LEVs as antibacterial therapeutics.

## 4. Materials and Methods

### 4.1. Cell Cultures and LEVs Isolation

For this work, we used NK-92 (ATCC, Manassas, VA, USA) cells, reproducing all the main morphological, phenotypic, and functional characteristics of activated NK cells. Cells were cultured according to the manufacturer’s instructions (ATCC, USA). Cell viability during cultivation and in the experiment was controlled using trypan blue; it was at least 95%.

Also, we used ESKAPE group bacteria: *Enterococcus faecium* (19434), *Staphylococcus aureus* (29213), *Klebsiella pneumoniae* (13883), *Acinetobacter baumannii* (19606), *Pseudomonas aeruginosa* (27853) and *Enterobacter* spp. (13047) (ATCC, USA) and cultured them on agarose medium, under appropriate biosafety containment, in accordance with institutional safety protocols for handling pathogenic microorganisms.

NK-92-derived LEVs were isolated using the standard differential centrifugation technique [21]. The day before LEV’s isolation, the culture medium in vials with NK-92 cells was completely changed, bringing the volume to 40 mL and the cell concentration to 4 × 10^5^ in 1 mL. Next, the contents of the vials were sequentially centrifuged at 200× *g* for 600 s and then at 9900× *g* for 600 s. The precipitate was removed, and the supernatant was centrifuged several times in a centrifuge cooled to 4 °C at 19,800× *g* for 1200 s, each time precipitating and concentrating LEVs. To control the size and stability of isolated LEVs, a Zetasizer NanoZS laser correlation spectrometer (Malvern Instruments, Malvern, UK) was used, a granulometric analysis was performed, and a zeta potential measurement was conducted.

### 4.2. Evaluation of TLR2 and TLR5 Expression on NK-92 Cell-Derived LEVs

LEVs were isolated as described in Section 4.1. For surface receptor analysis, LEVs were stained with fluorophore-conjugated monoclonal antibodies against TLR2 and TLR5 (BioLegend, San Diego, CA, USA), alongside appropriate isotype controls. Staining was performed according to the manufacturer’s protocol and analyzed on a BD FACSCanto II flow cytometer (BD Biosciences, Franklin Lakes, NJ, USA).

### 4.3. Quantification of Defensin-α1 in NK-92 Cells and Derived LEVs

Defensin-α1 levels in the conditioned medium of NK-92 cells and NK-92-derived LEVs were quantified using a commercial enzyme-linked immunosorbent assay kit (Cloud-Clone Corp., Wuhan, China), according to the manufacturer’s instructions. Conditioned media were collected from NK-92 cultures following incubation with bacterial supernatants or inflammatory inducers as described previously [20]. Samples were added to pre-coated plates, and the assay was carried out in accordance with the manufacturer’s instructions. Optical density was measured at 450 nm using a microplate spectrophotometer (Labsystems, Vantaa, Finland). Defensin-α1 concentrations were calculated from a standard curve.

### 4.4. Assessment of Bacterial Colony Formation and Growth in the Presence of NK-92 Cells or Derived LEVs

Bacteria and NK-92 cell cultures were subcultured according to standard protocols 24 h prior to the experiment. On the day of assay, bacterial suspensions were adjusted to 0.5 × 10^6^ CFU/mL in meat-peptone broth. For co-culture experiments, 50 µL of bacterial suspension and 50 µL of NK-92 cell suspension (0.5 × 10^5^ cells/mL) were combined in 96-well plates. In experiments with LEVs, they were isolated as described in Section 4.1, and the used concentration was 640 µg/10^6^ bacteria. Control wells contained bacteria alone in 100 µL total volume. At time points T^0^ (immediately after mixing) and T^1^ (1 h incubation at 37 °C), cultures were plated on a Petri dish with agar using a standard microbiological loop. After 24 h incubation at 37 °C, colony numbers were quantified using an automated colony counter, Scan500 (Interscience, France).

### 4.5. Confocal Microscopy Visualization of NK-92-Bacteria Interactions

Bacterial and NK-92 cell cultures were prepared as described in Section 4.4. For imaging, bacteria were labeled with CFSE (Sigma-Aldrich, Saint Louis, MA, USA) according to the manufacturer’s protocol. NK-92 cells were stained with APC-Cy7-conjugated anti-CD45 antibody (BD Biosciences, USA) and counterstained with DAPI (ServiceBio, Wuhan, China) for nuclear visualization. Labeled cells were mixed and incubated for 120 min at 37 °C in a humidified 5% CO_2_ atmosphere. Samples were mounted using antifade reagent (ServiceBio, China) and imaged on a NEXCOPE NCF950 laser scanning confocal microscope (Nexcope, Ningbo, China).

### 4.6. Flow Cytometry-Based Tracking of CFSE Label Transfer from NK-92-Derived LEVs to K. Pneumoniae and S. aureus

Flow cytometry-based tracking of CFSE label transfer from NK-92-derived LEVs to bacteria was evaluated using a modified protocol based on [40]. Twenty-four hour prior to LEVs’ isolation, NK-92 cells (3.2 × 10^7^) were labeled with CFSE (Sigma-Aldrich, USA) according to the manufacturer’s protocol or left unlabeled. The next day, LEVs derived from unlabeled and CFSE-labeled cells were isolated (int_LEVs and CFSE_LEVs, respectively). *K. pneumoniae* or *S. aureus* (1 × 10^6^ CFU/mL) were co-incubated with int_LEVs or CFSE_LEVs for 60 min at 37 °C. As a control, bacteria were also stained with CFSE (Sigma-Aldrich, USA), according to the manufacturer’s description; some of the bacteria were left unstained. Samples were analyzed by flow cytometry, bacterial fluorescence was evaluated by comparing it with negative (bacteria unstained with CFSE—unst) and positive (bacteria stained with CFSE—CFSE) controls.

### 4.7. S. aureus Viability Assessment Following LEVs Exposure

The impact of NK-92-derived LEVs on *S. aureus* viability was evaluated using the LIVE/DEAD BacLight Bacterial Viability Kit (Thermo Fisher Scientific, Waltham, MA, USA) according to the manufacturer’s instructions. Briefly, bacterial suspensions (1 × 10^6^ CFU/mL) were incubated with LEVs or intact for 60 min at 37 °C. Cells were then stained with SYTO 9/propidium iodide (PI) mixture for 15 min in the dark and immediately analyzed by flow cytometry. PI fluorescence was quantified in the PE channel.

### 4.8. Assay for S. auereus Antibiotic Susceptibility Modulation by NK-92-Derived LEVs

Antibiotic susceptibility testing was performed using the disk diffusion method. Bacterial suspensions (0.5 × 10^6^ CFU/mL in MPB) were pre-incubated with NK-92-derived LEVs for 60 min at 37 °C or intact. After they were plated on a Petri dish. Commercial antibiotic disks with clindamycin, erythromycin, cefoxitin, ampicillin/sulbactam, norfloxacin, and benzylpenicillin (Saint-Petersburg Pasteur Institute, Saint-Petersburg, Russia) were applied. Plates were incubated for 18–20 h at 37 °C, after which inhibition zone diameters were measured using an automated system Scan500 (Interscience, France). For clindamycin, results were cross-validated using E-test strips (bioMérieux, Marcy-l’Étoile, France).

## 5. Conclusions

This study provides preliminary insights into the functional properties of NK cells and their derived LEVs. Under controlled in vitro conditions, NK-92 cells and NK-92-derived LEVs exhibited a plausible antimicrobial effect against selected ESKAPE pathogens. Of particular note is the novel finding that NK-92-derived LEVs potentiate *S. aureus* susceptibility to clindamycin, evidenced by reduced minimum inhibitory concentrations. Packaging of defensin-α1 packaged into LEVs is suggestive of mediating antibacterial properties. Furthermore, the modulation of defensin secretion by inflammatory cytokines suggests that LEVs’ proteomic composition can be engineered through parental cell stimulation. Collectively, these findings highlight the potential of NK cell-derived LEVs as promising strategies to combat multidrug-resistant bacterial infections.

## Figures and Tables

**Table 1:** Modulation of antibiotic susceptibility in *S. aureus* by NK-92 cell-derived LEVs.

Antibiotic	Inhibition Zone Diameter (mm)							
	*S. aureus*	*S. aureus* *+*LEVs	*S. aureus*	*S. aureus* *+*LEVs	*S. aureus*	*S. aureus* *+*LEVs	*S. aureus*	*S. aureus* *+*LEVs +TNFα
Clindamycin	31.2	32.3	30.8	31.8	35	38	30	31
Erythromycin	30.7	31.1	29.7	30.8	30	32	30	32
Benzylpenicillin	25.2	24.6	24.8	24.9	25	25	25	25
Norfloxacin	22.7	23.3	23.3	24.7	30	26	24	25
Ampicillin/sulbactam	28	28	27.3	28.7	30	30	28	27
Cefoxitin	27.5	29.3	27.3	28.4	27	28	27	28

The table is based on 3 experiments with 2 technical repeats; the median value for every experiment is shown. An experiment with LEVs derived from pre-stimulated NK-92 is conducted once due to no effect and the high price of LEVs isolation in this case.

## Data Availability

The original contributions presented in this study are included in the article/Supplementary Material. Further inquiries can be directed to the corresponding author.

## Supplementary Materials

The following supporting information can be downloaded at: https://www.mdpi.com/article/10.3390/ijms27093953/s1.
