# Supplementary figures and images for: Modulation of ESKAPE Bacteria Properties by NK-92 and NK-92-Derived LEVs: First Insights
Source: Int J Mol Sci. 2026 Apr 29;27(9):3953. doi: 10.3390/ijms27093953 (PMC13164268; doi:10.3390/ijms27093953)

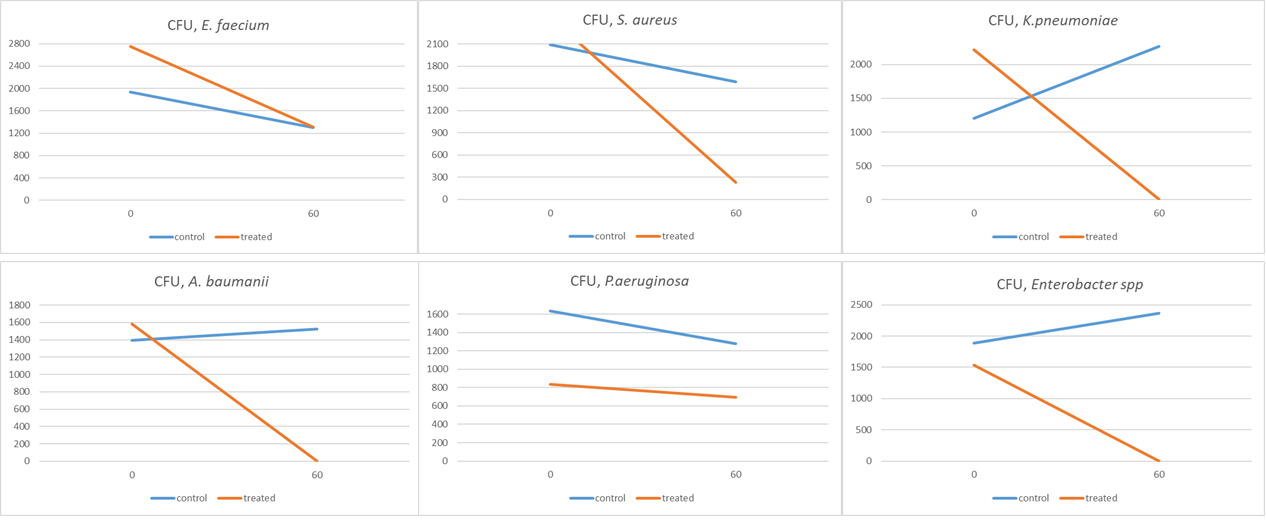

Supplement: Supplementary file 1 [file ijms-27-03953-s001.zip › Figure S1.TIF]

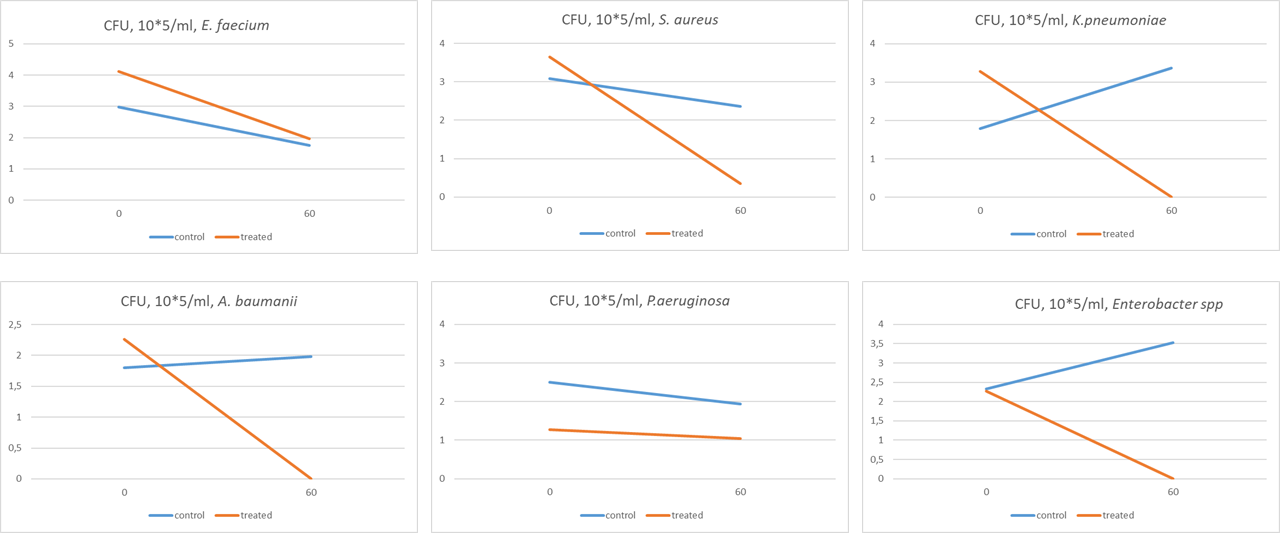

Supplement: Supplementary file 1 [file ijms-27-03953-s001.zip › Figure S2.TIF]

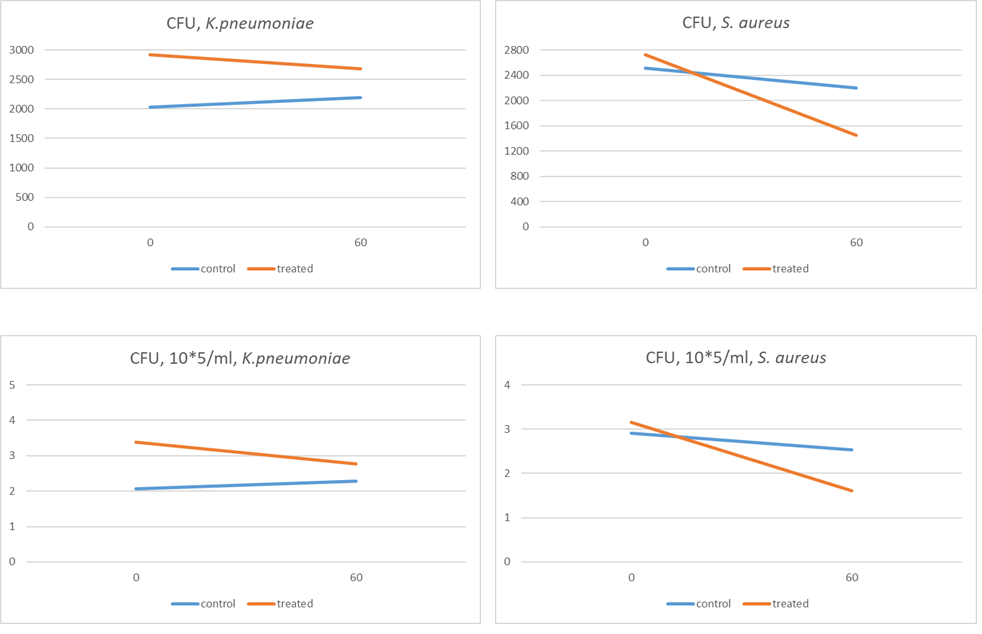

Supplement: Supplementary file 1 [file ijms-27-03953-s001.zip › Figure S3.TIF]

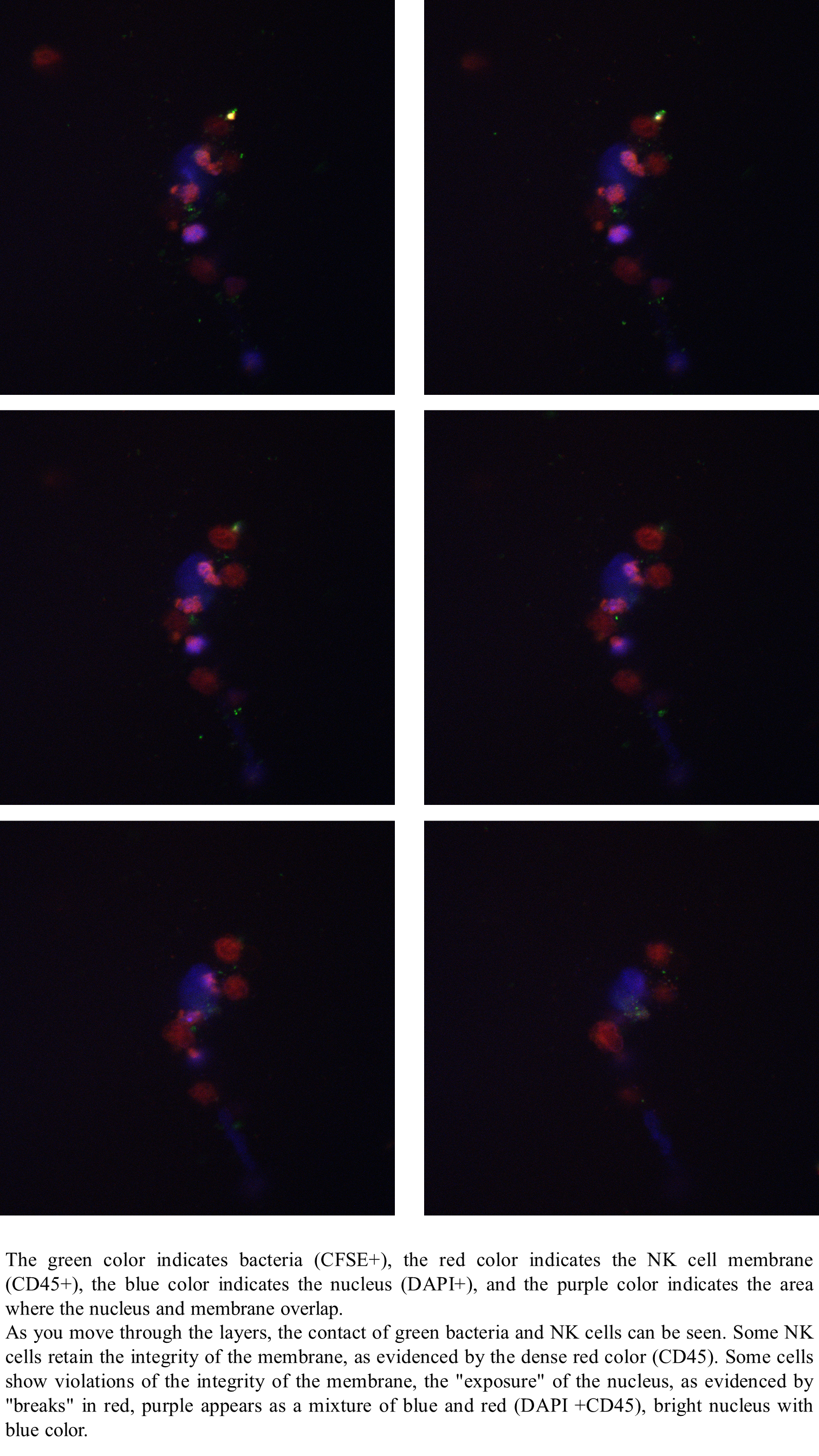

Supplement: Supplementary file 1 [file ijms-27-03953-s001.zip › Figure S4.tif]
